# Identification of a Novel SARS-CoV-2 Strain with Truncated Protein in ORF8 Gene by Next Generation Sequencing

**DOI:** 10.21203/rs.3.rs-413141/v1

**Published:** 2021-04-21

**Authors:** Stephanie DeRonde, Hannah Deuling, Jayme Parker, Jack Chen

**Affiliations:** University of Alaska Fairbanks

**Keywords:** Next-Generation Sequencing (NGS), COVID-19, SARS-CoV-2, Open Reading Frame (ORF)

## Abstract

Using next generation sequencing technology, we identified a truncated protein mutation located in the ORF8 gene which is near the end of the genome from nucleotides 27,878 to 27,958. The mutation in this novel strain created a stop codon and translates to the novel truncated ORF8 protein, creating a much smaller protein than most other strains of SARS-CoV-2. The novel truncated mutation is most closely related to nine SARS-CoV-2 strains found in Washington state. Our results show a novel strain of SARS-CoV-2 with a truncated ORF8 gene. This shortens the translated ORF8 protein. The effects of ORF8 protein and its functions are still uncertain but a truncated ORF8 could affect antibody response, severity of infection and inflammatory response.

## Introduction

At the end of 2019, severe acute respiratory syndrome coronavirus 2 (SARS-CoV-2) was identified in Wuhan, China as the cause of COVID-19 disease. Since then, the virus has spread to a global level causing 57,882,183 confirmed COVID-19 infections and 1,377,395 deaths as of November 22nd 2020 [10]. Symptoms of COVID-19 include fever, cough, shortness of breath and difficulty breathing among other symptoms. Severe cases of COVID-19 can result in respiratory disease and pneumonia requiring hospitalization [1]. It was declared a global pandemic in March 2020 by the World Health Organization and since has caused a negative effect on the health, socio-economics and the lives of billions of people [5].

SARS-CoV-2 is one of seven coronaviruses found to infect humans. It is an enveloped virus of single stranded RNA that codes for 6 major open reading frames (ORFs); ORF1a, ORF1b, spike (S), envelope (E), membrane (M) and nucleocapsid (N) among a few smaller ORF [6]. The genome sequence of SARS-CoV-2 was published early during COVID-19 pandemic, which shows that it is most closely related to a bat coronavirus - RaTG13 [11]. This led researchers to believe that it most likely got transferred from a bat through an unknown intermediate source to humans at the Hunan seafood market in Wuhan city of China [8]. SARS-CoV-2 has spread further and impacted more people than any other coronavirus. Knowing its genome structure is important for understanding why it has such a high transmission ability compared to other coronaviruses.

We have been working on sequencing positive COVID-19 specimens from across Alaska. We are sequencing the SARS-CoV-2 genome using Next Generation Sequencing technology in order to get the full genome coverage of the virus in each sample. Understanding the genomic structure of this virus is key for tracing the spread of the virus and used to see if and how the virus has mutated in Alaska and what strains are circulating in the state.

As the viral structure of SARS-CoV-2 has been better understood, we know that the genome encodes polyproteins pp1ab, four structural proteins and six accessory proteins; 3a, 6, 7a, 7b, 8, and 10 [2]. ORF8 is an accessory protein that is still being understood with its function in regard to the infectability of SARS-CoV-2. During sequencing of positive COVID-19 specimens, we found a novel SARS-CoV-2 strain with truncated mutation in the ORF8 gene. ORF 8 gene encodes for the ORF8 protein that is previously shown to affect immune response in humans [7]. Changes in the ORF8 protein may affect the infective properties of this particular strain of SARS-CoV-2. Understanding if this is a random mutation or a case of adaptive evolution of the virus is crucial when looking at creating a productive vaccine. One study found that one of the functions of the ORF8 gene could be coding proteins that are associated with viral replication. This could mean a truncated mutation may decrease replication in the host and affect invasion of host immune response. Another potential function of ORF8 is immune modulation through hijacking the host ubiquitin proteasome system (UPS) [7]. This could help the virus get through the host immune surveillance system and start proliferating inside humans. Overall, we need further research into the functional properties of the ORF8 protein and how it could be affected when truncated.

## Materials And Methods

### Clinical specimens.

Positive COVID-19 samples were collected in Alaska State Public Health Laboratories from testing centers around the state and frozen in a −80°C freezer until ready for sequencing. This research project was reviewed and approved by the University of Alaska Fairbanks Institutional Review Board (IRB) (Approval letter No. 667418–4). All methods were performed in accordance with the relevant guidelines and regulations.

### Construction of next generation sequencing libraries for Illumina.

The protocol followed was lab developed test (LDT) - Construction of Next Generation Sequencing Library for Illumina Sequencer Version 11. Only samples with a CT value under 20 were used for sequencing in order to increase likelihood of getting a full genome. The NEBNext Ultra II RNA Library Prep Kit for Illumina (New England Biolabs, Cat. No. NEB #E7770) was used to construct sequencing library.

RNA fragmentation and priming were completed by adding 13ul of each sample RNA to primers and loading in a thermal cycler. Samples underwent first strand cDNA synthesis by adding NEBNext First Strand Synthesis enzyme mix and then second strand cDNA synthesis through adding buffer and Second Strand Synthesis enzyme mix in thermal cycler. To purify the double-stranded cDNA, 144ul of AMPure XP beads were added to second strand cDNA synthesis and transferred to magnetic rack. After 3 minutes the supernatant was discarded without disturbing the beads that contained the DNA target. 80% ethanol was added and removed to remove residue and clean the beads. The beads were left for 3minutes for air dry instead of the protocol’s suggested 5 minutes because the reaction was performed in Fairbanks where the air is drier than most places. Next, 0.1X TE buffer was added to the dried beads then 50 μl of the supernatant was removed and transferred to a clean nuclease-free PCR tube. Some samples were stored at −20°C overnight at this point.

To complete end repair/dA-tail of cDNA library, NEBNext Ultra II End Repair Reaction buffer and enzyme mix were added to cDNA and incubated in thermal cycler. To perform adaptor ligation, NEBNext Ligation Enhancer, Adaptor (15 μM) and Blunt/TA Ligase Master Mix were added to samples while in thermal cycler. To purify the ligation reaction, AMPure XP beads were added and put on a magnetic rack. The same process as previously to clean the beads was performed. When done, 15 μl of the supernatant was transferred to a clean PCR tube to complete PCR enrichment of adaptor ligated DNA. To do this, NEBNext Ultra II Q5 Master Mix and Universal PCR Primer (10 μM) were both added to samples and put in a thermal cycler.

Next, AMPure XP Beads were added to purify the PCR reaction. The same process as previously to clean the beads was performed and then 20 μl of the supernatant was transferred to a clean 1.5 ml tube. To pool NGS library, 5 μl of each individual NGS library was taken and pooled into one 1.5 ml tube. 0.8X volume of resuspended Agencourt AMPure XP Beads were added to the tube and then the same process as previously to clean the beads was performed. At the end, 20 μl of the supernatant was transferred to a clean 1.5 ml tube.

### Sequencing run at Illumina MiSeq System.

To assess library quality and library pool, 2 μL of final NGS Library Pool was placed on Qubit Bioanalyzer® using Agilent DNA 1000 Kit. Based on results from Qubit, the final NGS Library Pool concentration was diluted to 4nM. For the final step and to prepare sequencing library, 4.5 μL of pure H_2_O, 0.5 μL of the 2 N NaOH, 4.5 μL of the 4-nM final NGS Library Pool and 0.5 μL of the 4 nM PhiX were all added to PCR tube and put in thermal cycler. Finally, 10 μL of the denatured library was transferred into the 990 μL HT1 buffer, resulting in 20 pM denatured Library/PhiX with10% PhiX library.

NGS Reagent Kit was thawed and brought to the Illumina MiSeq along with the sequence library. The protocol to start the MiSeq was followed then 600 μL of the Library/PhiX was loaded on the cartridge to start sequencing run.

## Results

**Figures 1:**
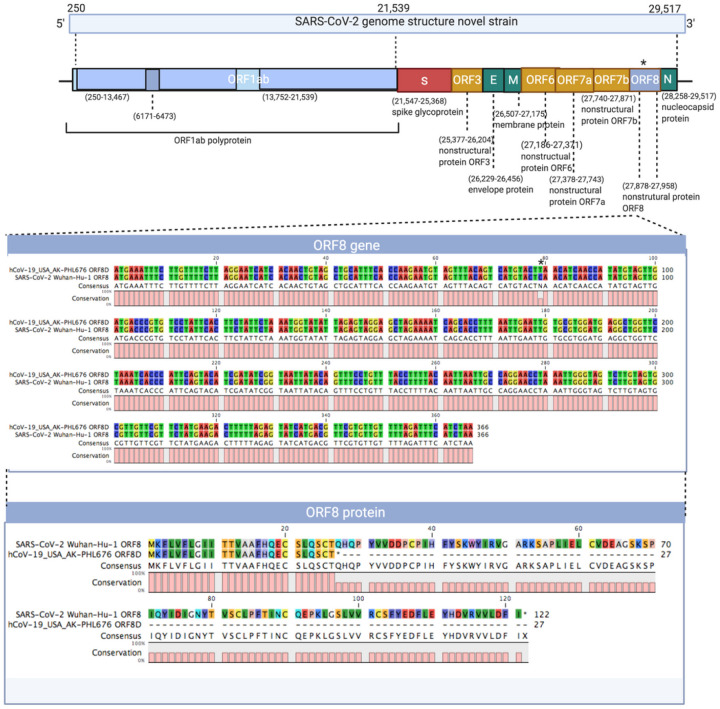
Genomic structure of novel SARS-CoV-2 strain. ORF8 gene sequences is enlarged and shown along with ORF8 amino acid sequences for both SARS-CoV-2 Wuhan-Hu-1 reference sequence and AK-PHL676 truncated mutation strain. * indicates where the point mutation occurred creating the truncation in the ORF8 protein. Created with BioRender.com

**Figures 2:**
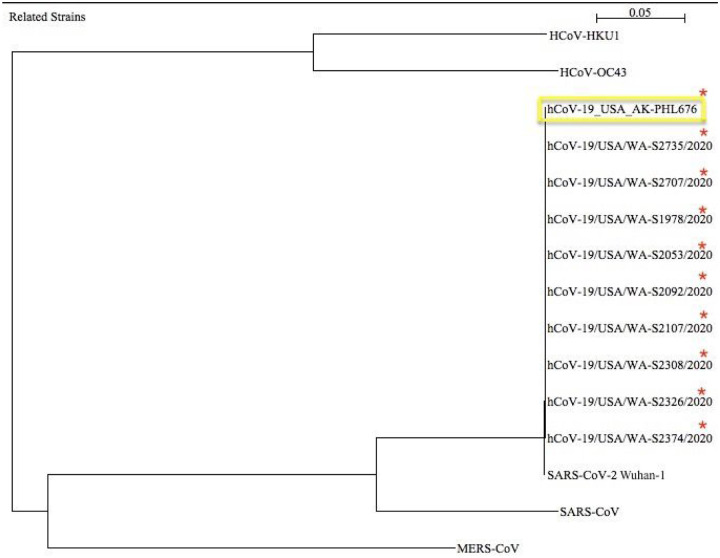
Phylogenetic tree of most closely related strains. The strain we sequenced was AK-PHL676 with the truncated ORF8 mutation highlighted in yellow. A red star indicates strains that have the same truncated mutation. A distance scale bar represents the number of differences between sequences (e.g. 0.05 means 5 % differences between two sequences. Tree was constructed using maximum likelihood method.

As shown in Fig. 1, the truncated mutation is located in the ORF8 gene which is near the end of the genome from nucleotides 27,878 to 27,958. The normal ORF8 gene is from nucleotides 27,878 to 28,246 so this novel strain has a truncation of 288 nucleotides. It was a point mutation from a C to a T creating a premature stop codon. The reference sequence SARS-CoV-2 Wuhan-Hu-1 shows the wild type gene length which translates to the ORF8 protein. The mutation in novel strain created a stop codon and translates to the novel truncated ORF8 protein, creating a much smaller protein than most other strains of SARS-CoV-2. A maximum likelihood phylogenetic tree of the most likely closely related stains was created in order to try to identify where this novel Alaskan strain came from (Fig. 2). The novel truncated mutation is most closely related to nine SARS-CoV-2 strains found in Washington state. These nine Washington have the same truncated mutation as the one that was found in the strain AK-PHL676 sequenced in Alaska. The original SARS-CoV-2 Wuhan-Hu-1 reference sequence does not have the truncated mutation and the phylogenetic tree shows that different SARS-CoV-2 strains have evolved from when the reference sequence was found in January 2020 in Wuhan, China. SARS-CoV, also known as SARS-1 is next most closely related to the truncated strain and then MERS-CoV. HCov-HKUI and HCoV-OC43 are least closely related to the truncated strain found in Alaska.

**Figures 3:**
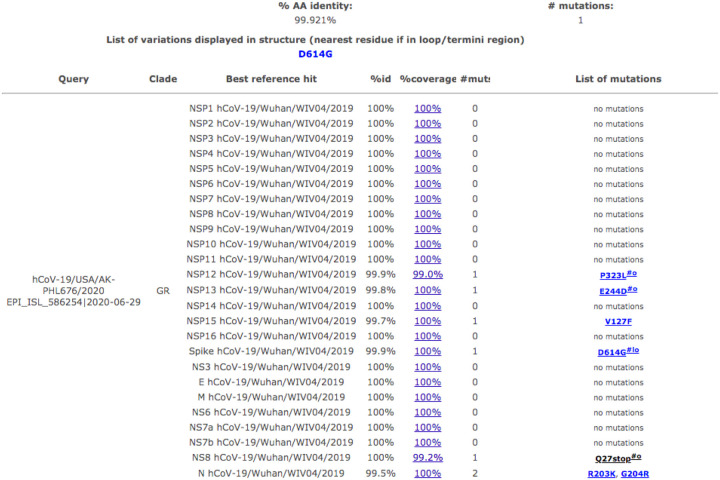
Related strains identified elsewhere. Related strains to Ak-PHL676 shown with list of mutation and variations in structure. Created through GISAID-CoVsurver-Developed by A*STAR Bioinformatics Institute, Singapore.

**Figures 4:**
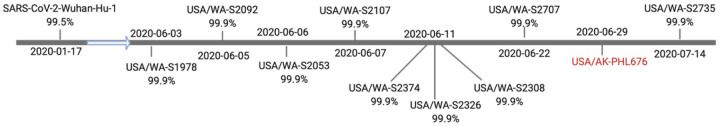
Timeline and evolution of related SARS-CoV-2 strains. USA/AK-PHL676 highlighted in red to show when it was found in Alaska compared to Washington strains. Eight strains were identified with the ORF8 mutation in Washington before it was discovered in Alaska. Percent identify to USA/AK-PHL676 is shown for each strain.

As shown in Fig. 3, the AK-PHL676 strain was shown to have related strains without the truncated mutation in the ORF8 gene. 25 strains were identified as related strains with no mutation or a mutation different than the one found in the ORF8 gene. There were six strains that were related to AK-PHL676 that showed variations displayed in the structure of their genome. Timeline and evolution of related SARS-CoV-2 strains show when USA/AK-PHL676 was found in Alaska compared to other related Washington strains (Fig. 4). Eight strains were identified with the ORF8 mutation in Washington before it was reported in Alaska on 2020-06-29. Percent identify to USA/AK-PHL676 is shown for each strain in Fig. 4.

## Discussion

Our results show a novel strain of SARS-CoV-2 with a truncated ORF8 gene. This shortens the translated ORF8 protein. The effects of ORF8 protein and its functions are still uncertain but a truncated ORF8 could affect antibody response, severity of infection and inflammatory response. This confirmed truncation of 288 nucleotides from the ORF8 region could affect how the human host reacts to this strain of SARS-CoV-2. One study found a 382-nucleotide deletion in the ORF8 gene [9]. They then studied different mutations and deletions in the ORF8 gene and looked at how it would affect ORF8 protein function. It was found that strains with significantly shorter ORF8 proteins had a higher replicative fitness as well as a heightened antibody response [9]. This deletion has been seen in many strains but the effects are not concrete yet. This study compared hospitalized patients with COVID-19 who either had the wild type ORF8 gene or the deletion in the ORF8 gene. They found that development of hypoxia requiring supplemental oxygen was less frequent in the group with the deletion in the ORF8 gene [12]. This could indicate that the truncation in the ORF8 could be associated with milder infections. It has also been suggested that the emergence of ORF8 deletions may be due to immune-driven selection [9]. As this virus continues to replicate in new hosts, we expect to see more mutations in the ORF8 region due to both random mutation and selection.

Our results from identifying the genomic organization of this novel Alaskan strain and then comparing it to the closely related strains show that it is most likely that this strain came from Washington State. The maximum likelihood phylogenetic tree shows that there are nine strains identified in Washington that all have the same truncated ORF8 gene mutation and are most closely related to this Alaskan novel strain. We have not yet discovered another sequence in Alaska with the same truncation however, it is possible that we will in the future since we have many more positive COVID-19 samples to sequence. It would be probable that Alaska has similar strains that are circulating in Washington due to the high level of connection between Seattle and Alaskan airport travelers.

With SARS-CoV-2 spreading rapidly across the globe, understanding the genome sequence of different strains has become increasingly important. Different studies have looked at the significance of mutations in many SARS-CoV-2 strains. One study did an analysis of 2,492 complete genomes and found of those there were 1,516 different nucleotide-level variations at different positions [4]. This leads to the understanding that there are many variations in the SARS-CoV-2 genome and an increasing amount as the virus continues to infect more people. The heterogeneity of this virus needs to be better understood when looking at the implications of mutations and how they can affect immune response in humans along with the severity of the disease. Understanding the differences in mutated protein structure is important when trying to create a lasting vaccine that will protect against SARS-CoV-2 as it continues to mutate in the population.

Understanding the genomic makeup of SARS-CoV-2 is essential as we combat the effects of the COVID-19 pandemic. Knowing which strains and what mutations may be circulating in the state of Alaska provides information into how infectious the virus is and how it may be evolving. This study states that ORF8 is a rapidly evolving accessory protein that has been proposed to interfere with immune responses [3]. If we can increase whole genome sequencing in order to gain a better understanding of the genomic makeup of this virus, the next step will be to better understand each protein’s role in the evolution of SARS-CoV-2 and how a mutation could change that role. If we can confirm that a mutation in ORF8 changes the immune response of the virus then we are better equipped to deal with the pandemic.

## Conclusions

Our results show a novel strain of SARS-CoV-2 with a truncated ORF8 gene. This shortens the translated ORF8 protein. The effects of ORF8 protein and its functions are still uncertain but a truncated ORF8 could affect antibody response, severity of infection and inflammatory response.
